# Two novel variants in *DYRK1B* causative of AOMS3: expanding the clinical spectrum

**DOI:** 10.1186/s13023-021-01924-z

**Published:** 2021-06-30

**Authors:** Elvia C. Mendoza-Caamal, Francisco Barajas-Olmos, Elaheh Mirzaeicheshmeh, Ian Ilizaliturri-Flores, Carlos A. Aguilar-Salinas, Donaji V. Gómez-Velasco, Isabel Cicerón-Arellano, Adriana Reséndiz-Rodríguez, Angélica Martínez-Hernández, Cecilia Contreras-Cubas, Sergio Islas-Andrade, Carlos Zerrweck, Humberto García-Ortiz, Lorena Orozco

**Affiliations:** 1 Clinical Area, National Institute of Genomic Medicine, SS, Mexico City, Mexico; 2 Immunogenomics and Metabolic Diseases Laboratory, National Institute of Genomic Medicine, SS. Periférico Sur 4809, Colonia Arenal Tepepan, Alcaldía Tlalpan, C.P. 14610, Mexico City, Mexico; 3 SEPI-UPIIH, National Polytechnic Institute, Pachuca, Hidalgo, Mexico; 4 Metabolic Diseases Research Unit, National Institute of Medical Science and Nutrition Salvador Zubirán, Mexico City, Mexico; 5 Department of Endocrinology and Metabolism, National Institute of Medical Science and Nutrition Salvador Zubirán, Mexico City, Mexico; 6 Direction of Nutrition, National Institute of Medical Science and Nutrition Salvador Zubirán, Mexico City, Mexico; 7 School of Medicine and Health Sciences, Monterrey Institute of Technology, Mexico City, Mexico; 8 Integral Clinic of Surgery for Obesity and Metabolic Diseases, General Hospital Tláhuac, SS, Mexico City, Mexico

**Keywords:** AOMS3, Diabetes, *DYRK1B*, Metabolic syndrome, Monogenic, and Obesity

## Abstract

**Background:**

We investigated pathogenic *DYRK1B* variants causative of abdominal obesity-metabolic syndrome 3 (AOMS3) in a group of patients originally diagnosed with type 2 diabetes. All *DYRK1B* exons were analyzed in a sample of 509 unrelated adults with type 2 diabetes and 459 controls, all belonging to the DMS1 SIGMA-cohort (ExAC). We performed in silico analysis on missense variants using Variant Effect Predictor software. To evaluate co-segregation, predicted pathogenic variants were genotyped in other family members. We performed molecular dynamics analysis for the co-segregating variants.

**Results:**

After filtering, Mendelian genotypes were confirmed in two probands bearing two novel variants, p.Arg252His and p.Lys68Gln. Both variants co-segregated with the AOMS3 phenotype in classic dominant autosomal inheritance with full penetrance. In silico analysis revealed impairment of the DYRK1B protein function by both variants. For the first time, we describe age-dependent variable expressivity of this entity, with central obesity and insulin resistance apparent in childhood; morbid obesity, severe hypertriglyceridemia, and labile type 2 diabetes appearing before 40 years of age; and hypertension emerging in the fifth decade of life. We also report the two youngest individuals suffering from AOMS3.

**Conclusions:**

Monogenic forms of metabolic diseases could be misdiagnosed and should be suspected in families with several affected members and early-onset metabolic phenotypes that are difficult to control. Early diagnostic strategies and medical interventions, even before symptoms or complications appear, could be useful.

**Supplementary Information:**

The online version contains supplementary material available at 10.1186/s13023-021-01924-z.

## Background

Abdominal obesity-metabolic syndrome 3 (AOMS3 [

OMIM:615812]) is a rare autosomal dominant disorder caused by pathogenic variants in the dual-specificity tyrosine phosphorylation-regulated kinase 1B gene (*DYRK1B*) located on chromosome 19q13.2 [1]. This monogenic form of metabolic syndrome (MetS) is characterized by abdominal obesity, type 2 diabetes, hypertension, and early-onset coronary artery disease [2]. DYRK1B inhibits the Sonic Hedgehog and WNT pathways, increasing the expression of master adipogenic transcription factors CCAAT/enhancer binding protein (C/EBP-alpha) and peroxisome proliferator-activated receptor gamma (PPAR-gamma) [2]. Moreover, DYRK1B induces the expression of glucose-6-phosphatase, a key enzyme in hepatic gluconeogenesis [3].

The identification of carriers of pathogenic variants in genes such as *DYRK1B* could be useful for establishing early diagnostic strategies and medical interventions in a reasonable number of affected individuals even before symptoms or complications appear. Until now, only two different mutations (p.Arg102Cys and p.His90Pro) in *DYRK1B* have been described as being causative of AOMS3 in three Iranian families and five unrelated Caucasian individuals [2]. However, families with rare monogenic forms of metabolic diseases could be misdiagnosed or even overlooked if causative variants are not directly explored, especially in populations with a high prevalence of these entities.

Therefore, we searched for *DYRK1B* variants in the exome sequencing data derived from 968 unrelated individuals (509 with type 2 diabetes) belonging to the DMS1 SIGMA-cohort (ExAC) [4], focusing on variants classified by Variant Effect Predictor (VEP) as deleterious and damaging to confirm their co-segregation with AOMS3. Here, we describe two novel *DYRK1B* mutations as causative of AOMS3 in two families previously misdiagnosed with type 2 diabetes.

## Results

### Identification and co-segregation of pathogenic variants in the DYRK1B gene

**Fig. 1:**
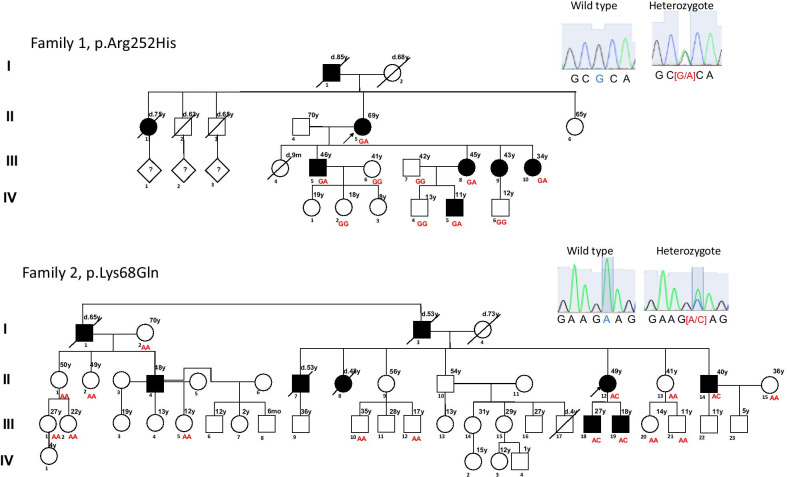
Pedigree of two families with an autosomal dominant inheritance pattern of *DYRK1B* mutations associated with AOMS3. Family 1 with p.Arg252His (Arg: C**G**C/His: C**A**C). Family 2 showing the p.Lys68Gln mutation (Lys: **A**AG/Gln: **C**AG). The index cases (II.5 in family 1 and II.12 in family 2) are indicated by arrows. Genotypes are presented under each individual. Family members with clinical signs compatible with AOMS3 are indicated by solid symbols. Slashes indicate that the individual is deceased

Of the 968 unrelated individuals, 52.6% (n = 509) had type 2 diabetes according to the American Diabetes Association criteria [5]. The remaining 459 individuals were healthy subjects > 45 years old with fasting glucose (FG) levels < 100 mg/dL. We were able to identify 29 variants in *DYRK1B* (Additional file 1: Table S1), including seven missense variants and four variants predicted to be deleterious or damaging by VEP (Table 1). Two of these latter variants, p.Leu28Pro and p.Asp436Asn, were found in six heterozygote individuals (n = 5 and n = 1, respectively), all of them without type 2 diabetes or clinical characteristics of AOMS3. The remaining two missense variants, p.Arg252His and p.Lys68Gln, were found in two unrelated individuals manifesting symptoms suggestive of AOMS3, such as childhood-onset abdominal obesity, type 2 diabetes, hypertriglyceridemia, and arterial hypertension. Both of these individuals also had a family history of obesity and type 2 diabetes, as well as premature death secondary to myocardial infarction in first and second-degree relatives. We were able to recruit three generations of these two families with 26 traceable members, 9 of whom were *DYRK1B* pathogenic-variant carriers (5 harbored the p.Arg252His variant and 4 p.Lys68Gln). The pedigree analysis revealed co-segregation of the *DYRK1B* genotype with the AOMS3 phenotype, showing a characteristic autosomal dominant inheritance pattern with full penetrance (Fig. 1) and age-dependent variable expressivity. None of the non-carrier family members had type 2 diabetes or previous diagnosis of dyslipidemia, and only one had arterial hypertension. The carriers had severe metabolic compromise characteristic of AOMS3 (Table 2), and we observed a significant difference between carriers and non-carriers of pathogenic variants; carriers had higher body mass index (BMI), FG, and triglyceride levels. Furthermore, abdominal obesity started in childhood and progressed to morbid obesity in youth. The current mean BMI was 36.6 Kg/m^2^, but all patients had a higher BMI (mean 45.5 Kg/m^2^) before the diagnosis of type 2 diabetes. HOMA-IR and the Matsuda index (normal > 3) were impaired in all carriers, even in a child with normoglycemia (Additional file 1: Table S2). Similarly, the mean age of diagnosis of type 2 diabetes was 34 years old, and most of them had difficult to control disease (mean HbA1c: 87 mmol/mol (10.1%) and mean FG: 191.1 mg/dL). The mean serum triglyceride levels were 315.9 mg/dL, though all adult carriers underwent pharmacological treatment for dyslipidemia, except individual II.14 in family 2, who had serum triglyceride levels of 650 mg/dL.

**Table 1:** Variant Effect Predictor analysis of missense variants in *DYRK1B*

Variant	Nucleotide change	Amino acid change	SIFT^a^	PolyPhen2
rs746933234	c.755G > A	p.Arg252His	Deleterious (0.02)	Probably damaging (0.996)
rs373850179	c.202A > C	p.Lys68Gln	Deleterious (0.01)	Possibly damaging (0.713)
rs34587974	c.83T > C	p.Leu28Pro	Deleterious (0.01)	Possibly damaging (0.563)
rs752428936	c.1306G > A	p.Asp436Asn	Deleterious (0.05)	Probably damaging (0.957)
rs148788670	c.1733C > T	p.Pro578Leu	Tolerated-low confidence (0.23)	Benign (0)
rs144370928	c.209G > A	p.Arg70Gln	Tolerated (0.06)	Benign (0.047)
rs771417583	c.1666A > C	p.Thr556Pro	Deleterious-low confidence (0)	Benign (0)

^a^*SIFT* sorting tolerant from intolerant

**Table 2:** Clinical characteristics of the 26 individuals genotyped in two families

Family/mutation		ID	Gender	Age (years)	OB/Age of onset (years)	BMI (Kg/m^2^)	BMI before T2D^†^	T2D/Age of diagnosis (years)	FG (mg/dL)	HbA1c (%)	Previous diagnosis of Dyslipidemia/Age of diagnosis (years)	TG (mg/dL)	Chol (mg/dL)	HDL (mg/dL)	HTN/Age of diagnosis (years)	SBP (mmHg)	DBP (mmHg)
F1/p.Arg252His	Non-carriers	IV.6	M	12	No	24	NA	No	89	–	No	145	174	35	No	115	71
		IV.4	M	13	No	19.7	NA	No	83	5.3	No	86	110	41	No	114	58
		IV.2	F	18	No	26.4	NA	No	83	5.3	No	109	209	73	No	104	68
		III.7	M	42	Yes/42	31.6	NA	No	68	–	No	118	192	34	No	127	83
		III.6	F	41	Yes/41	32.5	NA	No	67	5.5	No	83	143	31	No	110	71
	Carriers	IV.5	M	11	Yes/Childhood	30	NA	No	98	5.5	No	130	138	49	No	108	65
		III.10	F	34	Yes/Childhood	35.6	35.6	Yes/34	168	9.1	Yes/29	479	179	23	No	123	87
		III.8	F	45	Yes/Childhood	36.7	42.3	Yes/43	165	7.7	Yes/44	412	192	36	No	106	66
		III.5	M	46	Yes/Childhood	41.5	47.8	Yes/41	120	6.8	Yes/30	341	146	39	Yes/41	107	70
		II.5	F	69	Yes/Childhood	33.8	35.7	Yes/41	124	8.9	Yes/62	249	241	57	Yes/62	145	72
	Average ± SD carriers with T2D	–	–	48.5 ± 14.7	–	36.9 ± 3.2	40.3 ± 5.8*	39.7 ± 3.9	144.2 ± 25.7*	8.1 ± 1.0*	41.2 ± 15.4	370.2 ± 98.5	189.5 ± 39.4	38.7 ± 14.0	51.5 ± 14.8	120.2 ± 18.2	73.7 ± 9.1
F2/p.Lys68Gln	Non-carriers	III.21	M	11	No	18.6	NA	No	86	–	No	48	190	48	No	106	85
		III.5	F	12	No	16.9	NA	No	75	–	No	75	132	38	No	106	65
		III.20	F	14	No	25.4	NA	No	88	–	No	83	223	69	No	109	75
		III.12	M	17	Yes/17	37	NA	No	90	–	No	106	211	32	No	126	79
		III.2	F	22	No	21.5	NA	No	82	–	No	59	171	40	No	99	60
		III.1	F	27	No	29.5	NA	No	75	–	No	87	168	41	No	102	43
		III.10	M	35	No	20.7	NA	No	81	–	No	56	135	41	No	112	70
		II.15	F	36	No	25.9	NA	No	94	–	No	79	107	40	No	117	79
		II.13	F	41	No	24.5	NA	No	77	–	No	66	214	76	No	110	62
		II.2	F	49	Yes/44	38.8	NA	No	109	6.1	No	105	131	29	Yes/44	132	87
		II.1	F	50	Yes/50	31.4	NA	No	89	–	No	85	174	48	No	134	76
		I.2	F	70	No	20.7	NA	No	85	–	No	75	203	71	No	122	72
	Carriers	III.19	M	18	No	26.4	NA	No	102	5.9	Yes/16	125	123	28	No	131	74
		III.18	M	27	Yes/Childhood	40.4	50.8	Yes/18	276	12.5	Yes/24	231	192	38	No	137	83
		II.14	M	40	Yes/Childhood	51.9	55.6	Yes/29	257	14.5	Yes/37	650	245	NA	Yes/40	149	87
		II.12	F	49	Yes/Childhood	33.4	50.8	Yes/31	228	11.2	Yes/31	226	166	42	Yes/45	128	64
	Average ± SD carriers with T2D	–	–	38.7 ± 11	–	41.9 ± 9.3	52.4 ± 2.8*	26 ± 7	253.6 ± 24.1*	12.7 ± 1.7*	30.67 ± 6.51	369 ± 243.3	201 ± 40.2	40 ± 2.8	42.5 ± 3.5	138 ± 10.5	78 ± 12.2

ID, identification; OB, obesity; BMI, body mass index; T2D, type 2 diabetes; FG, fasting glucose; HbA1c, glycosylated hemoglobin; TG, triglyceride; Chol, total cholesterol; HDL, high density lipoprotein; HTN, hypertension; SBP, systolic blood pressure; DBP, diastolic blood pressure; M, male; F, female; NA, not apply; †, referred value by the patient; SD, standard deviation.

*Adjusted *p*-*value* by gender < 0.05 when contrast average value of p.Arg252His with T2D and the average value of p.Lys68Gln with T2D

The clinical record documented that arterial hypertension was the last trait to become apparent, and it was present in 4 of 5 carriers older than 40 years old (the mean age of the diagnosis of arterial hypertension was 47 years). The pulse wave velocity increased in all carriers, even in the absence of arterial hypertension (Additional file 1: Table S2). p.Lys68Gln carriers had a higher BMI, lower insulin secretion, worse diabetes control, and an increased urine albumin/creatinine ratio than p.Arg252His carriers (Table 2 and Additional file 1: Figure S1). There were no differences in HDL, apolipoprotein B, creatinine, and hepatic enzymes levels between carriers and non-carriers. The uric acid level was elevated in two carriers, one with the p.Arg252His variant and another with p.Lys68Gln variant (Additional file 1: Table S2).

Interestingly, we were able to document metabolic disorders in two carrier males in their second decade of life. The youngest was an 11-year-old child who carried the p.Arg252His variant (IV.5 in family 1), had obesity (BMI: 30 Kg/m^2^, weight > 95th percentile of CDC grow charts), and demonstrated insulin resistance (HOMA-IR: 5.44) but not other metabolic abnormalities. The second carrier was an 18-year-old who carried the p.Lys68Gln variant (III.19 in family 2), was overweight (BMI: 26.4 Kg/m^2^, weight 90-95th percentile of CDC grow charts), serum FG 102 mg/dL, and hypertriglyceridemia since 16 years old; he was undergoing pharmacological treatment. Notably, both carriers had a strictly controlled diet because of the family history of type 2 diabetes.

### Molecular dynamics

**Fig. 2:**
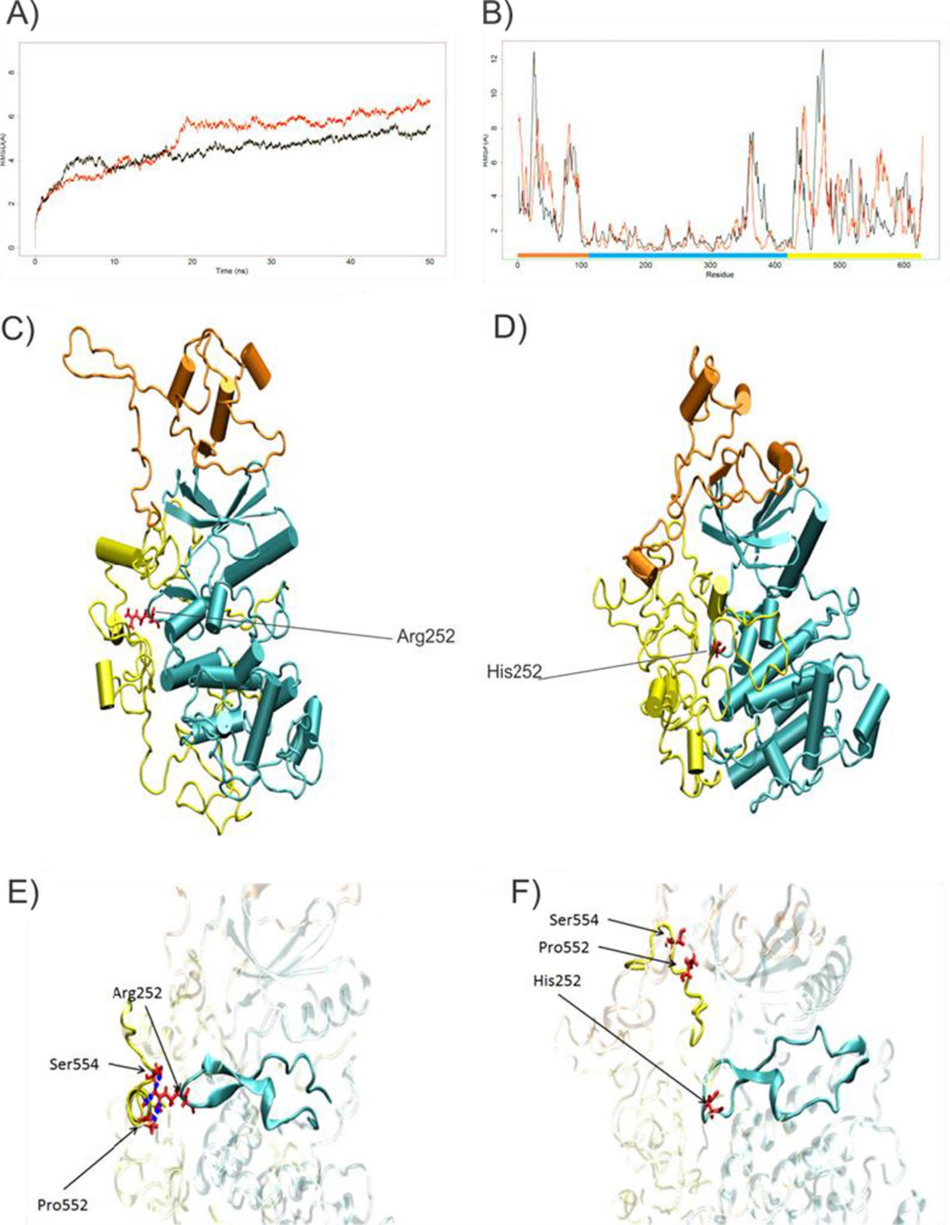
Molecular dynamic analysis of DYRK1B-252His. **a** Root mean square deviation (RMSD) of atomic positions, analysis of wild-type protein (black line), and DYR1B-252His (red line). **b** Root mean square fluctuation (RMSF) of the wild-type protein (black line) and DYR1B-252His (red line). **c** Wild-type structure. **d** Structure of DYRK1B-252His. The protein structure colors are the same as previously described. **e** Region of interest zoomed-in for Arg252. The hydrogen bonds are depicted by the blue dots. **f** Same region of interest with the His252 variant

To gain more insight into the effect of the variants on protein structure and function, we performed molecular dynamics (MD) simulation trajectory analysis of DYRK1B-252Arg and DYRK1B-252His (Fig. 2). The root mean square deviation (RMSD) values indicate that DYRK1B-252His took a longer period of time to reach equilibration (Fig. 2a). The root mean square fluctuation (RMSF) analysis documented instability and structural changes in the N- and C-terminal regions, possibly due to the lack of hydrogen bonds (Fig. 2b, brown and yellow boxes). In addition, analyses revealed that, in contrast, to the results when Arg was present at position 252, His252 lost the hydrogen bonds with Pro552 and Ser554, resulting in conformational changes in the catalytic site and potentially losing functionality (Fig. 2c–f).

Despite the DYRK1B-68Gln mutant showed no-structural changes and relative stability, in silico analysis, revealed that this mutation is located within the nuclear localization sequence (NLS) proposed by Jhaisha et al. (positions 66–86) [6], suggesting that, although its catalytic function could not be affected, it could accumulate in the cytosol.

## Discussion

Over the past few years, there have been many attempts to gain more insight into the genetic factors involved in metabolic diseases [7–9]. Multiple genetic variants have been shown to participate in the pathogenesis of each of the traits of metabolic syndrome. The polygenic nature of these conditions implies that the effect of the majority of genetic variants in these disorders is small [7]. However, families in which autosomal dominant inheritance is present have been used to search for rare mutations in genes with a strong contribution [10, 11]. The availability of exome sequencing is leading to the rapid identification of new players in the pathogenesis of metabolic diseases. This is the case for *DYRK1B* mutations, which cause a rare monogenic form of MetS known as AOMS3 [2]. This syndrome has been described as the presence of abdominal obesity, type 2 diabetes, hypertension, and early-onset coronary artery disease [2]. Pathological *DYRK1B* variants result in the enhanced expression of transcription factors C/EBPalpha and PPARgamma, leading to increased adipogenesis. In addition, *DYRK1B* increases glucose-6-phosphatase, which is strongly associated with insulin resistance, explaining the metabolic phenotypes characterizing AOMS3 [2, 3].

The participation of *DYRK1B* in MetS is poorly studied. Six years after *DYRK1B* was associated with AOMS3, only a few carriers have been reported [1]. Furthermore, only two missense *DYRK1B* mutations (p.Arg102Cys and p.His90Pro) have been identified in these individuals [2]. It is possible for rare diseases that mimic symptoms of common diseases to be confused with them. The prevalence of metabolic diseases in Mexico is one of the highest in the world [12, 13], and rare metabolic diseases are often hidden behind them; therefore, families with rare monogenic forms can remain unnoticed.

Next generation sequencing technologies have greatly improved the possibility of identifying rare pathogenic variants involved in monogenic diseases [4, 11]. In this study, after analyzing the sequence of all *DYRK1B* exons in a sample of 968 adult, including 509 with type 2 diabetes (SIGMA-ExAC) [14], we found 29 variants. SIFT and PolyPhen predicted that four of them (p.Leu28Pro, p.Asp436Asn, p.Arg252His, and p.Lys68Gln) have a deleterious and damaging effect. The p.Leu28Pro variant was described previously as having a protective effect against type 2 diabetes in a phenome-wide association study [15]. In this study, we found five heterozygotes individuals with neither type 2 diabetes nor AOMS3, but we were not able to recruit the familial relatives to confirm its protective effect. The p.Asp436Asn variant was found in a male heterozygote, who was 98 years old and metabolically healthy. However, p.Arg252His and p.Lys68Gln exhibited co-segregation with the AOMS3 phenotype with classic dominant autosomal inheritance and full penetrance. Both variants were absent in the 1000 Genomes database [16], and the gnomAD database reports eight individuals with p.Arg252His variant and five with p.Lys68Gln variant, including the ones we report here. In Latinos, these variants were found with a frequency of 0.00008677 and 0.00005783, respectively [17].

MD structural analyses predicted that, when DYRK1B-252His was present, the formation of three hydrogen bonds was impaired, with instability in the N- and C-terminal regions. In contrast, the p.Lys68Gln variant did not produce any significant changes in the protein structure, although the NLS motif could be affected, in accordance with Kosugi et al., who showed that the N-terminal basic pattern “Lys_68_Arg_69_” is required for a strong NLS activity [18]. More over, the effect of these variants could be similar to those documenting that DYRK1B-102Cys and DYRK1B-90Pro variants cause changes to the structure and perinuclear aggregation but barely affect the kinase activity [6]. We classified p.Arg252His and p.Lys68Gln as causative of AOMS3, in agreement with the American College of Medical Genetics and Genomics standards and guidelines [19].

Compared to non-carriers of pathogenic variants, we found that carriers had higher BMI, and FG and triglyceride levels. Furthermore, pulse wave velocity was increased in all carriers, even in the absence of arterial hypertension, explaining the high cardiovascular risk found in this condition. Insulin action was decreased in all but one case, but the insulinogenic index was significantly decreased in all carriers, even in normoglycemic individuals, suggesting that the remarkable severity of hyperglycemia found in this condition results from a combination of moderate insulin resistance and a moderate to severe defect in insulin secretion. This could be supported by recent findings showing that DYRK1A and DYRK1B play an important role in human β cells proliferation [20].

Notably, we are describing additional features of the disease and manifestations that develop throughout life. Our findings exhibit age-dependent variance in expressivity in all patients, with some clinical features apparent at a very early age and other manifestations appearing later in life. Central obesity and insulin resistance started during childhood and then progressed rapidly to morbid obesity and labile type 2 diabetes. They also had severe hypertriglyceridemia with onset as a teenager. Similarly, AOMS3 patients developed hypertension in the fifth decade of life, and both families had a history of premature death due to cardiovascular events. Another interesting finding was that p.Lys68Gln carriers had a higher BMI, hypoinsulinemia, worse diabetes control and an increased urine albumin/creatinine ratio compared to the p.Arg252His carriers, suggesting an allelic heterogeneity.

Remarkably, none of the non-carriers had diabetes; only a 49-year-old woman (II.2 in family 2) had glucose serum levels of 109 mg/dl, along with obesity and hypertension. Other non-carrier individuals also had low levels of HDL or obesity, however, none of them met all the AOMS3 clinical features. These manifestations could be a reflection of the high prevalence of metabolic diseases in our population [12, 13].

## Conclusions

How can these findings improve public health? AOMS3 should be suspected in individuals with the clinical outcome described above and a family history of affected first degree relatives. Assessment of the first-degree relatives should be performed routinely in subjects with extreme obesity. An autosomal dominant inheritance pattern should be systematically sought. Identifying variants involved in rare disorders could bring to light new genes associated with common diseases and may have implications for screening and targeted therapy. Otherwise, cascade genetic screening increases the possibility of finding pre-symptomatic carriers and lead to the implementation of strategies to prevent or delay the disease course, benefiting a reasonable number of affected families. The large number of cases and remarkably large structure of the Mexican families are unique opportunities to identify new genetic variants in understudied populations. Special care should be taken with the youngest members of the families, in whom cardiovascular prevention should be implemented early in life.

## Material and methods

### Study participants

We included 968 unrelated adult Mexican Mestizos belonging to the DMS1 SIGMA-cohort [14] who were previously sequenced by Sure-Select Human All Exon v2.0 (Illumina) and included in the ExAC project [4]. A peripheral blood sample was collected after fasting for at least 8 h. The following clinical and biochemical data were obtained for all participants of the DMS1 cohort using the Synchron CX5 Analyzer System (Beckman Coulter Fullerton, CA, USA): FG (mg/dL), HDL (mg/dL), and serum triglycerides (mg/dL). HbA1c levels were measured using the IN2it analyzer (Bio-Rad, Hercules, CA, USA). Blood pressure was measured using a digital blood pressure monitor (HEM-907XL, OMRON). Weight and height were measured using a body composition monitor (HBF-500 INT, OMRON) and electronic stadiometer (ADE Germany). Waist circumference was measured midway between the inferior margin of the ribs and the border of the iliac crest using a flexible clinical measuring tape.

The study was carried out according to the Declaration of Helsinki and was approved by the Research, Ethics, and Biosafety Human Committees of the Instituto Nacional de Medicina Genómica (INMEGEN) in Mexico City. All participants provided written informed consent. They were recruited from August 2017 to December 2018, all of them inhabited the Valley of Mexico.

### Identification of pathogenic variants

We identified *DYRK1B* variants by analyzing the exon sequences in each individual. To find the deleterious and damaging variants, we annotated them using the VEP toolset [21]. Missense variants that were predicted as deleterious or affecting the protein structure were used to perform genotype–phenotype linkages. Individuals identified as carriers of these variants and had clinical manifestations suggesting AOMS3 were re-contacted and invited, along with their family members, to participate in a familial co-segregation study. All individuals who participated in the family segregation study provided written informed consent; in the case of children, the parents provided written consent and the children assented.

Genotyping was performed by Sanger sequencing using specific primers: p.Arg252His forward primer, CCTTTCTTCTCTGGCCAT; p.Arg252His reverse primer, ACCCAAACTACTAGCCGTGC; p.Lys69Gln forward primer, TGCCAGCAGCCTTACAGTT; p.Lys69Gln reverse primer, CCACTGCGCAACGATGTAGTC. The obtained sequences were analyzed by 4Peaks V1.8 [22].

The same clinical, demographic, and biochemical data described for index cases were obtained for each family participant. In addition, seven carriers (four p.Arg252His and three p.Lys69Gln) and three non-carriers were clinically and biochemically re-evaluated at the Unidad de Investigación de Enfermedades Metabólicas, Instituto Nacional de Ciencias Médicas y Nutrición. The assessment included fasting biochemical measurements (i.e., clinical chemistry, liver panel, and lipid profile), estimation of body composition, pulse wave velocity measurements, and albumin/creatinine ratio in a spot urine sample. An oral glucose tolerance test was performed using a 75 g oral glucose charge. Serum glucose and insulin were measured at fasting, 30-, 60-, 90-, 120-, and 180-min after oral glucose intake. The glucose concentration was measured by an automated glucose analyzer (Yellow Springs Instruments Co.). The serum insulin concentration was measured by a chemiluminescent immunoassay (Beckman Coulter Access 2) and HbA1c levels by HPLC (Variant II Turbo, BIORAD). Cholesterol, triglycerides, HDL, apolipoprotein B, uric acid, creatinine, and hepatic enzymes levels were measured using colorimetric assays (Unicel DxC 600 Synchron Clinical System Beckman Coulter). LDL was calculated by the Friedewald equation when the triglyceride concentration was < 250 mg/dL.

### Molecular dynamics

The wild-type amino acid sequence of DYRK1B (Q9Y463) from the UniProt database [23] was modeled to obtain the 3D protein structure using the I-TASSER server [24]. The structures of mutated proteins were predicted using the predicted wild-type protein and VMD v1.9.3 software [25]. Next, we carried out an atomistic MD simulation with explicit atom representation for proteins, water, and ions under force-field using the CHARMM package and NAMD v2.3 software [26]. Periodic boundary conditions, particle mesh Ewald, and a non-bonded cut-off of 14 Å and 2 fs time step were used. The isothermal-isobaric conditions were maintained with a Langevin thermostat (310 K) and Langevin piston barostat (1 atm). For each model, the system was subjected to energy minimization for 1000 steps, followed by equilibration for 5 ns, and the simulation continued for 50 ns without restraints. In the simulations analyses, we used the VEGAZZ v3.1.2 [27], Carma [28], and R cran project v3.4 [29] programs. The MD analysis included the RMSD and the RMSF.

### Statistical analysis

Clinical data are reported as mean ± standard deviation. Data were analyzed using R cran project v3.4 [29] and a general linear model test for comparing metabolite levels between carriers and non-carriers. Gender- and age-adjusted *P* < 0.05 was considered significant.

## Supplementary Information

**Additional file 1. Figure S1**. Glucose tolerance curves. **Table S1**. Variants on DYRK1B in a sample of 968 Mexican Mestizos. **Table S2**. Pulse wave velocity and other biochemical studies.

## Data Availability

The datasets generated and analysed during the current study are not publicly available due this work is part of a larger project but are available from the corresponding author on reasonable request.

## Abbreviations

AOMS3: Abdominal obesity-metabolic syndrome 3
DYRK1: *B* Dual-specificity tyrosine phosphorylation-regulated kinase 1B
C/EBPalpha: Transcription factors CCAAT/enhancer binding protein
FG: Fasting glucose
INMEGEN: Instituto Nacional de Medicina Genómica
MD: Molecular dynamics
MetS: Metabolic syndrome
NLS: Nuclear localization signal
PPAR-gamma: Peroxisome proliferator-activated receptor gamma
VEP: Variant Effect Predictor
RMSD: Root mean square deviation
RMSF: Root mean square fluctuation
