# Supplementary material for: Two novel variants in DYRK1B causative of AOMS3: expanding the clinical spectrum
Source: Orphanet J Rare Dis. 2021 Jun 30;16:291. doi: 10.1186/s13023-021-01924-z (PMC8247206; doi:10.1186/s13023-021-01924-z)
Supplement: Supplementary file 1 — Additional file 1. Figure S1. Glucose tolerance curves. Table S1. Variants on DYRK1B in a sample of 968 Mexican Mestizos. Table S2. Pulse wave velocity and other biochemical studies. [file 13023_2021_1924_MOESM1_ESM.pdf]

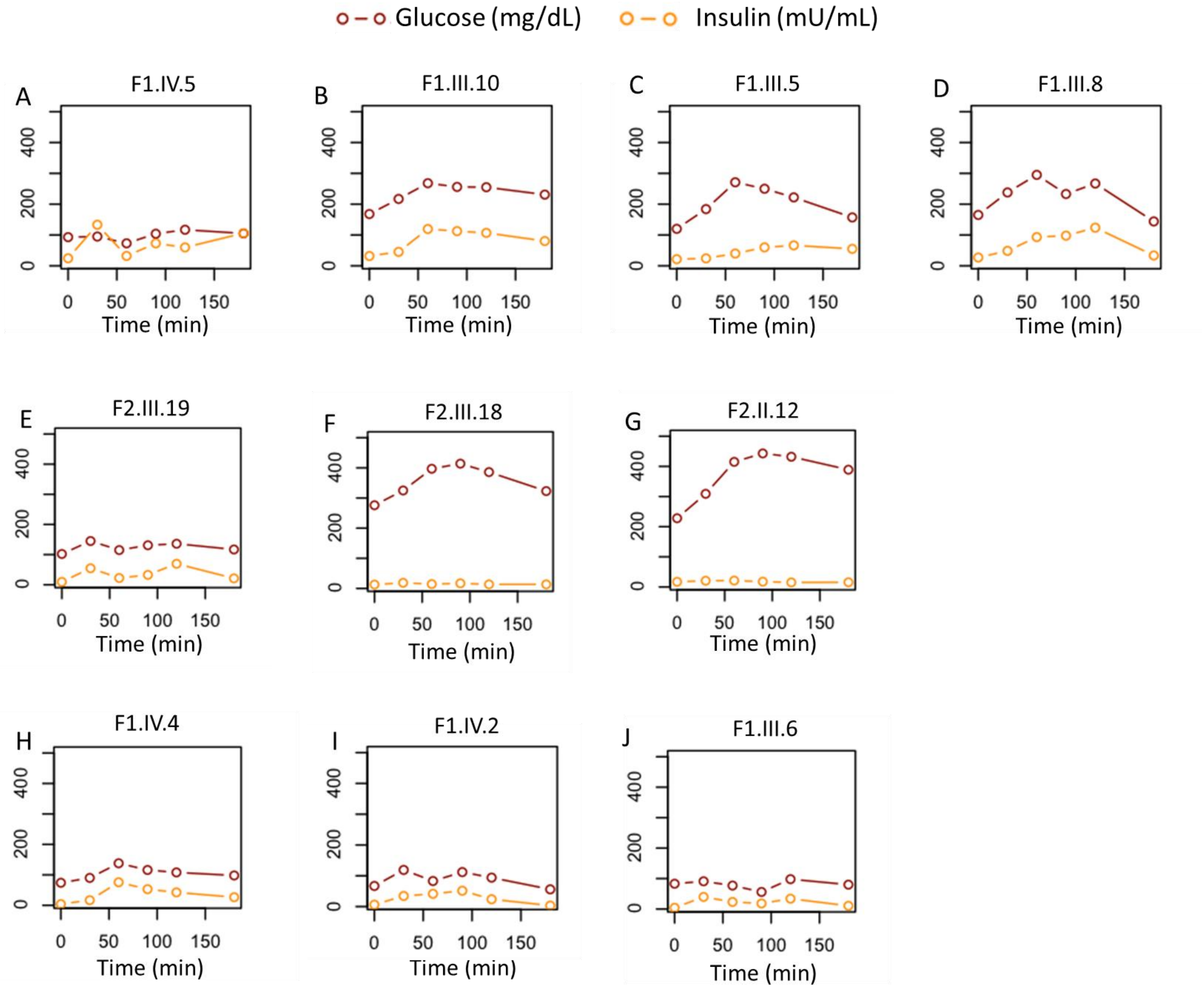

Supplemental Figure S1. Glucose tolerance curves. Blood glucose (brown) and plasma insulin levels (yellow) in response to a 75 g oral glucose charge measured at fasting, 30-, 60-, 90-, 120-, and 180-minutes after oral glucose intake. (A-D) Carriers of p.Arg252His, (E-G) carriers of p.Lys68Gln, and (H-J) non-carriers.

Supplemental Table S1. Variants on *DYRK1B* in a sample of 968 Mexican Mestizos.

Variant	Position	Allele Frequency % (allele number)	Major/minor allele	Annotation	Consequence
rs746933234	40318989	0.052 (1)	C/T	Missense	p.Arg252His
rs373850179	40321185	0.052 (1)	T/G	Missense	p.Lys68Gln
rs34587974	40321404	0.258 (5)	A/G	Missense	p.Leu28Pro
rs752428936	40317417	0.052 (1)	C/T	Missense	p.Asp436Asn
rs148788670	40316512	0.052 (1)	G/A	Missense	p.Pro578Leu
rs144370928	40321178	0.052 (1)	C/T	Missense	p.Arg70Gln
rs771417583	40316579	0.052 (1)	T/G	Missense	p.Thr556Pro
rs149054894	40321322	0.052 (1)	G/A	Synonymous	p.Thr55Thr
rs146996483	40318018	0.362(7)	G/A	Synonymous	p.Ala334Ala
rs779778950	40318985	0.052 (1)	G/A	Synonymous	p.Ser253Ser
rs759826670	40319144	0.052 (1)	G/C	Synonymous	p.Leu200Leu
rs758562354	40320638	0.052 (1)	C/T	Synonymous	p.Gln134Gln
rs538648311	40321054	0.052 (1)	G/A	Synonymous	p.Tyr111Tyr
rs371524346	40321087	0.052 (1)	G/A	Synonymous	p.Ile100Ile
rs370695515	40317421	0.103 (2)	C/T	Synonymous	p.Thr434Thr
rs751446560	40316679	0.207 (4)	G/A	Synonymous	p.Pro522Pro
rs765204866	40320714	0.103 (2)	G/C	Non coding transcript exon	NA
rs56077108	40319227	2.738 (53)	C/T	Splice region	NA
rs201207939	40318138	0.052 (1)	G/A	Intron	NA
rs377517922	40316949	0.052 (1)	G/A	Intron	NA
rs147939499	40316754	0.052 (1)	T/C	Intron	NA
rs76318664	40319232	0.155 (3)	G/A	Intron	NA
rs112350522	40319260	0.258 (5)	C/T	Intron	NA
rs118167426	40318132	0.620 (12)	C/T	Intron	NA
rs56110449	40318087	28.822 (558)	G/A	Intron	NA
rs772833215	40316742	0.052 (1)	A/G	Intron	NA
rs780638095	40318314	0.052 (1)	G/A	Intron	NA
rs755640697	40318332	0.052 (1)	C/T	Intron	NA
rs769863099	40316954	0.052 (1)	G/T	Intron	NA

NA: Not apply

Supplemental Table S2. Pulse wave velocity and other biochemical studies.

Variant status	ID	PWV	AORTIC AU	AIx	AIx_75%	LDL-c (mg/dL)	Uric Acid (mg/dL)	CR-S (mg/dL)	ALT (IU/L)	AST (IU/L)	GGT (IU/L)	APO_B (mg/dL)	ACR (mg/dL)	HOMA-IR	Matsuda Index	Insulinogenic Index
p.Arg252His	IV.5	4.3	15	31	32	63	7.7	0.7	13	22	13	59.8	-	5.61	2.58	54.37
	III.10	7.1	12	28	28	-	4.1	0.54	55	43	30	108.1	18.7	13.19	0.95	0.27
	III.8	6.1	25	45	43	-	3.9	0.62	24	25	25	109.9	1.8	10.96	1.07	0.29
	III.5	6.6	15	47	42	-	5.4	0.88	54	47	38	69.2	4.9	6.34	2.06	0.04
p.Lys68Gln	III.19	5.6	4	9	12	70	6.5	0.75	22	22	22	65.6	-	2.36	5.03	1.05
	III.18	6.6	6	15	17	108	5.4	0.55	25	23	30	110.4	163.1	8.72	2.2	0.12
	II.12	6.3	23	47	43	79	8	0.95	21	21	16	89.2	252.4	9.62	1.9	0.04
F1. non-carrier	IV.4	3.6	6	19	12	52	5.8	0.8	35	32	17	52.8	-	0.72	8.6	0.8
	IV.2	4.7	14	35	26	114	3	0.63	16	21	8	83.9	-	0.84	12.21	4.45
	III.6	5.4	20	53	50	95	3.1	0.42	12	17	6	79.5	-	1.03	8.28	0.54

ID, identification; PVW, pulse wave velocity; AU, augmentation; AIx, augmentation index; AIx_75%, corrected augmentation index to 75%; LDL-c, low density lipoprotein cholesterol; CR-S, creatinine serum; ALT, alanine aminotransferase; AST, aspartate aminotransferase; GGT, gamma glutamine transferase; APO_B, apolipoprotein B; ACR, urine albumin/creatinine ratio; HOMA-IR; homeostatic model assessment for insulin resistance.
